# Circulating zinc-α2-glycoprotein levels are low in newly diagnosed patients with metabolic syndrome and correlate with adiponectin

**DOI:** 10.1186/s12986-017-0210-6

**Published:** 2017-08-14

**Authors:** Lu Lei, Kejia Li, Ling Li, Xia Fang, Tingting Zhou, Cheng Zhang, Yong Luo, Hua Liu, Xiaoqiang Li, Hongting Zheng, Lin Zhang, Gangyi Yang, Lin Gao

**Affiliations:** 1 Department of Endocrinology, the Affiliated Hospital, Zunyi Medical College, Guizhou, China; 2 Department of Endocrinology, the Second Affiliated Hospital, Chongqing Medical University, Chongqing, China; 3 Key Laboratory of Diagnostic Medicine (Ministry of Education) and Department of Clinical Biochemistry, College of Laboratory Medicine, Chongqing Medical University, Chongqing, China; 4 The Center of Clinical Research of Endocrinology and Metabolic Diseases in Chongqing and Department of Endocrinology, Chongqing three Gorges Central Hospital, Chongqing, China; 5 Department of Pediatrics, University of Mississippi Medical Center, 2500 North State Street, Jackson, Mississippi MS 39216-4505 USA; 6 Children’s Hospital of Chongqing Medical University, Chongqing, China; 7 Department of Endocrinology, Xinqiao Hospital, Third Military Medical University, Chongqing, China; 8 Department of Endocrinology, the Ba Yan NaoEr Hospital, Inner Mongolia, 015000 China

**Keywords:** Zinc-α2-glycoprotein, Adiponectin, Metabolic syndrome, Insulin resistance

## Abstract

**Background:**

Zinc-α2-glycoprotein (ZAG) is a novel adipokine that reduces insulin resistance, protecting against type 2 diabetes. However, past studies have been contradictory. This cross-sectional study aims to investigate the association of circulating ZAG with metabolic syndrome (MetS) in middle-aged and older Chinese adults.

**Methods:**

Four hundred eighty nine individuals (234 healthy controls and 255 MetS patients) were examined. All individuals were screened for MetS according to the diagnostic guidelines of the United States National Cholesterol Education Program (NCEP) Expert Panel Adult Treatment Panel (ATP) III criteria. Circulating ZAG and ADI levels were measured by ELISA. Blood fat, glucose and insulin were measured with a commercial kit. Circulating ZAG levels were compared with various parameters in study subjects.

**Results:**

Plasma ZAG levels were lower in MetS patients compared to those of the healthy controls (35.0 ± 11.7 vs. 46.1 ± 18.6 mg/L, *P* < 0.01). ZAG showed a positive correlation with age, HDL-C, HOMA-β and ADI, but a negative correlation with Fat%, BMI, WHR, blood pressure, triglycerides, FFA, FBG, 2 h–BG, fasting insulin, 2 h–Ins, HbA1c and HOMA-IR (*P* < 0.01). When the population was divided according to tertiles of ADI, subjects in the highest tertile had the highest ZAG levels. The analysis of ROC curves revealed that the best cutoff value for plasma ZAG to predict MetS was 45.2 mg/L (sensitivity 92%, specificity 59%, and AUC 0.80).

**Conclusions:**

We found that circulating ZAG levels were decreased in patients with MetS. In fact, circulating ZAG decreased progressively with an increasing number of MetS components and associated with ADI levels, suggesting that ZAG is related to IR and MetS and may be a sensitizer.

**Trial Registration:**

ChiCTR-OCC-11001422. Registered 23 June 2011.

## Background

Metabolic syndrome (MetS) is a clustering of cardiovascular risk factors including visceral obesity, disturbances of carbohydrate and lipid metabolism, and hypertension. The cardiovascular risk is proportional to the number of MetS components [1]. People diagnosed with MetS are known to be more susceptible to developing type 2 diabetes mellitus (T2DM) and face a 2-fold increased risk for cardiovascular disease (CVD) [2, 3]. MetS patients are also more susceptible to developing hypertension and cancers [4–6]. A meta-analysis has shown that the rate of cardiovascular disease, coronary heart disease (CHD) and stroke in MetS subjects is 50% higher than in healthy controls [4]. Although extensive research efforts have been put forth to elucidate the pathogenesis of MetS, it is still largely unknown. In recent years, some adipocyte-related cytokines have been shown to be biomarkers relative to MetS such as adiponectin (ADI). Insulin resistance (IR), the consequence of visceral obesity, has been known as a major pathomechanism in the development of MetS components [7]. The changes of circulating levels in some cytokines may indicate a potential role for MetS pathogenesis.

Zinc-α2-glycoprotein (ZAG) is a 40-kD soluble glycoprotein isolated from human plasma [8] and has also been found in other secretions, such as saliva, milk, sweat, urine, and cerebrospinal fluids [9]. One study showed that ZAG is also an adipokine because it is secreted by animal adipose tissues [10]. Although the biological functions of ZAG are largely unknown, it has been shown that ZAG is a novel adipokine and that its expression in adipose tissue is down-regulated in obese mice [10]. In addition, it has also been demonstrated that ZAG knockout mice are susceptible to weight gain when fed a high fat diet (HFD) which is associated with decreased lipolysis [11]. Importantly, Balazet et al. reported that silencing ZAG led to a reduction of ADI, insulin receptor substrate-1(IRS-1) and glucose transporters-4 (GLUT4) gene/protein expression in primary human adipocytes indicating that ZAG plays a pivotal role in regulating adipose tissue and whole-body insulin sensitivity [12].

In a human study, it has been demonstrated that ZAG is negatively relative to body weight and induces lipolysis in obese patients [13]. Recently, we have reported that circulating ZAG levels are markedly lower in patients with newly diagnosed type 2 diabetes mellitus (nT2DM) than in healthy controls. Circulating ZAG positively correlated with plasma ADI levels and negatively correlated with obesity related parameters and homeostasis model assessment of insulin resistance (HOMA-IR) [14]. ADI is an adipocyte-secreted hormone that has important associations with obesity and insulin resistance. A strong positive correlation between circulating ZAG and ADI suggests that ZAG may also be an insulin sensitizer. More recently, we have shown that circulating ZAG levels were much lower in women with polycystic ovary syndrome (PCOS) and IR than in healthy women and positively correlated with M-value, determined by an euglycemic-hyperinsulinemic clamp (EHC) considered the ‘gold standard’ measure of insulin sensitivity [15]. Therefore, ZAG has been considered to be a new candidate for involvement in the pathogenesis of IR and dysmetabolism. However, previous studies in humans and rodents have produced conflicting results regarding the link between ZAG and IR, T2DM, and obesity [9–15]. Currently, only one study has addressed the association between circulating ZAG and MetS [16]. As with most new discoveries, this finding needs to be reproduced and further investigated. The aim of the current study is to investigate the association between the number and type of MetS components and circulating ZAG levels in an elderly population. In addition, the relationship between circulating ZAG levels and ADI is also investigated in MetS patients.

## Methods

### Participants

Four hundred eighty nine individuals, 255 subjects with MetS (aged 39–82, male/female 125/130) and 234 healthy controls (aged 37–80, male/female 124/110), were recruited for this study from outpatients attending the Internal Medicine Department or routine medical check-up at the Second Affiliated Hospital, Chongqing Medical University, or from the community or schools through advertisement during February 2014 to December 2015. The diagnosis of MetS was based on the United States National Cholesterol Education Program (NCEP) Expert Panel Adult Treatment Panel (ATP) III criteria [17]. MetS individuals have more than three of following characteristics: 1) central obesity (waist circumference exceeds 90 or 80 cm for Asian male and female, respectively); 2) hypertension(systolic pressure equals or exceed 130 mmHg or diastolic pressure equals or exceeds 85 mmHg);3) elevated blood glucose (fasting glucose level equals or exceeds5.5 mmol/L [100 mg/dL]), or T2DM; 4) elevated plasma triglycerides (TG; level equals or exceeds 1.69 mmol/L [150 mg/dL]) and 5) low level of high-density lipoprotein-cholesterol (HDL-C level equals or is less than 1.04 mmol/L[40 mg/dL] for maleand1.29 mmol/L[50 mg/dL] for female) are regarded as MetS positive. The exclusion criteria included cancer, current renal or liver disease, and a history of myocardial infarction, stroke, or transient ischemic attacks, or a history of regular use of medications for diabetes, hypertension, and/or lipid-lowering drugs. The patients with MetS were newly diagnosed and had not been treated with any agents. Age-matched healthy subjects without clinical evidence of major diseases were used as the controls.

Subjects were classified according to body mass index (BMI) (lean <25; and overweight/obese ≥ 25 kg/m^2^). Subjects were also divided into subgroup according to gender (male: *n* = 149; female: *n* = 240). All subjects gave their written informed consent before entering the study. This study was conducted in accordance with the Declaration of Helsinki and approved by the human research ethics committee of Chongqing Medical University.

### Anthropometric and biochemical measurements

Anthropometric measurements including height and body weight were taken. The BMI, waist circumference and waist-to-hip ratio (WHR) were calculated. HOMA-IR was calculated using the following eq. [18]: HOMA-IR = fasting insulin (FIns, mU/L) × fasting blood glucose (FBG, mmol/L)/22.5. The homeostasis model assessment of insulin secretion (HOMA-β) were calculated using the following equation: HOMA-β = [20 × FIns (μU/ml)]/[FBG (mmol/L) – 3.5]. Plasma was stored at − 80 °C for the determination of free fatty acid (FFA), insulin, ZAG, ADI and blood fat levels. Plasma glucose and glycosylated haemoglobin (HbA1c) were immediately measured by the glucose-oxidase method and anion-exchange HPLC, respectively. Plasma insulin levels were measured using chemiluminescence. Free fatty acids (FFAs) were measured with a commercial kit (Randox Laboratories Ltd., Antrim, UK). Total cholesterol, low-density lipoprotein cholesterol (LDL-C), TG, and HDL-C were determined enzymatically using an auto-analyzer (Hitachi 747; Hitachi, Tokyo, Japan).

### Measurements of plasma adipokines

Circulating ZAG concentration was measured with an ELISA obtained from Ray Biotech Inc. following the manufacturer’s protocol. The limit of detection was 0.02 mg/mL, and intra- assay and inter-assay variations were 2.56% and 6.63% respectively. Plasma ADI levels were also measured with an ELISA from Adipobiotech [19].

### Statistical analysis

All analyses were performed with SPSS version 15.0 (SPSS, Chicago, IL). Data are expressed as mean ± SD or median (interquartile range). Normal distribution of the data was tested using Kolmogorox-Smirnov test. Several variables were skewed and logarithmically transformed to obtain a normal distribution. Comparisons between groups were performed with Non-parametric tests or Student t test. Correlations between variables were assessed using Pearson correlation analyses by controlling for the covariates. Multiple linear regression was performed to determine variables that had independent associations with circulating ZAG, and included were all variables with significant associations or correlations with circulating ZAG and those with possible biological relevance. Multiple stepwise regression analysis was performed with age-adjusted plasma ZAG levels as the dependent variables, and by entering the independent variable with the highest partial correlation coefficient at each step, with a F-value probability for inclusion of 0.05 and 0.01 for removal. The trend of circulating ZAG levels associated with MetS was analyzed using the Row Mean Scores and Cochran-Armitage trend test. The ORs for ZAG levels and MetS were calculated by binary logistic regression. Receiver operator characteristic (ROC) curve analysis was employed to identify the optimal cut-off values of ZAG to diagnose MetS. *P* < 0.05 was considered significant.

## Results

### Characteristics and circulating ZAG levels of study participants

**Fig. 1:**
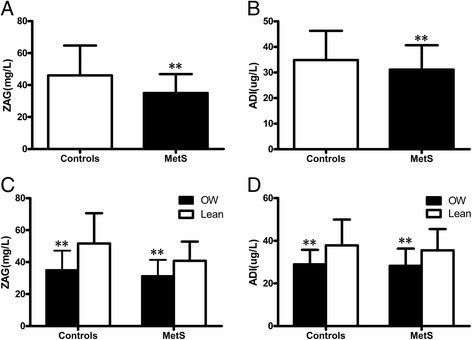
**a** Circulating ZAG levels in MetS patients and controls; **b** Circulating ADI levels in MetS patients and controls (vs. controls: **, *P* < 0.01); **c** Circulating ZAG levels in MetS patients and controls according to BMI; **d** Circulating ADI levels in MetS patients and controls according to BMI (lean: BMI < 25 kg/m^2^ and overweight/obese: BMI ≥ 25 kg/m^2^; vs. lean: **, *P* < 0.01). Overweight/obese: *n* = 156 for MetS; *n* = 79 for the controls; lean: *n* = 99 for MetS; *n* = 155 for the controls

Anthropometric and metabolic parameters of the study subjects were showed in Table 1. There were no significant differences in age, TC and LDL-C levels between control and MetS groups. However, as expected, BMI, the percentage of fat in vivo (FAT%), WHR, systolic blood pressure (SBP), diastolic blood pressure (DBP), TG, FFA, FBG, 2 h blood glucose after a 75 g glucose load (2 h–BG), FIns, 2-h plasma insulin after glucose overload (2-h Ins), HbA1c and HOMA-IR were significantly increased, whereas HDL-C and HOMA-β were significantly decreased in MetS subjects when compared with the controls (*P* < 0.05 or *P* < 0.01). Importantly, MetS patients had lower circulating ZAG and ADI levels than the control subjects (both *P* < 0.01; Fig. 1a and b). Furthermore, circulating ZAG levels were significantly lower in MetS with overweight/obese (*n* = 156) and control subjects with overweight/obese (*n* = 79) than their lean subjects (MetS, *n* = 99; control, *n* = 155) (MetS: 31.4 ± 10.1 vs. 40.8 ± 11.9 μg/L; Controls: 35.1 ± 12.0 vs. 51.6 ± 18.9, both *P* < 0.01) (Fig. 1c). Similar changes were found in circulating ADIlevels (MetS: 28.3 ± 8.1vs.35.5 ± 10.0 μg/L; Controls: 28.94 ± 6.82 vs. 37.9 ± 12.1, both *P* < 0.01) (Fig. 1d). Circulating ZAG levels were similar in both men and women (39.02 ± 16.48 vs. 41.76 ± 16.18 μg/L), whereas circulating ADI levels were higher in women than in men (36.06 ± 11.36 *vs.* 30.04 ± 9.05 μg/L, *P* < 0.01).

**Table 1:** Main clinical features and circulating ZAG levels in MetS and control subjects

Variable	Controls (*n* = 234)	Mets (*n* = 255)	*P*-value
male/female	124/110	125/130	NS
Age (year)	51.9 ± 9.2	52.4 ± 7.4	NS
BMI (kg/m2)	23.6 ± 3.5	26.1 ± 3.7	<0.001
FAT (%)	29.2 ± 6.9	30.6 ± 5.1	0.014
WHR	0.88 ± 0.06	0.91 ± 0.06	<0.001
SBP (mmHg)	119.6 ± 12.8	127.6 ± 13.2	<0.001
DBP (mmHg)	75.2 ± 7.5	78.0 ± 8.2	<0.001
TC (mmol/L)	4.88 ± 0.92	5.00 ± 0.63	NS
TG (mmol/L)	1.38 ± 0.62	2.07 ± 0.54	<0.001
HDL-C (mmol/L)	1.47 ± 0.34	1.32 ± 0.27	<0.001
LDL-C (mmol/L)	2.74 ± 0.76	2.72 ± 0.69	NS
FFA (μmol/L)	0.54 ± 0.27	0.67 ± 0.29	<0.001
FBG (mmol/L)	6.15 (5.30–8.57)	8.98 (8.12–9.89)	<0.001
2 h–BG (mmol/L)	9.52 (6.14–14.86)	16.86 (12.76–20.44)	<0.001
FIns (mU/L)	10.90 (7.00–13.86)	13.11 (9.76–16.47)	<0.001
2 h–Ins (mU/L)	40.89 (34.8–51.71)	45.73 (37.85–55.89)	0.011
HbA1c (%)	6.98 ± 1.52	7.84 ± 1.21	<0.001
HOMA-IR	3.08 (1.80–4.93)	5.45 (3.68–7.05)	<0.001
HOMA-β	60.76 (47.51–99.76)	50.44 (37.89–59.90)	<0.001
Adiponectin (μg/L)	34.9 ± 11.4	31.1 ± 9.5	<0.001
ZAG (mg/L)	46.1 ± 18.6	35.0 ± 11.8	<0.001

*MetS* metabolic syndrome, *BMI* Body mass index, *FAT%* the percentage of fat in vivo, *WHR* Waist hip ratio, *SBP* Systolic blood pressure, *DBP* Diastolic blood pressure, *TC* Total cholesterol, *TG* Triglyeride, *HDL-C* High-density lipoprotein cholesterol, *LDL-C* Low-density lipoprotein cholesterol, *FFA* free fatty acid, *FBG* fasting blood glucose, *2 h–BG* 2-h blood glucose after glucose overload, *FIns* fasting insulin, *2 h–Ins* 2-h plasma insulin after glucose overload, *HbA1c* glycosylated haemoglobin, *HOMA-IR* homeostasis model assessment of insulin resistance, *HOMA-β* HOMA β cell insulin secretion index

### Correlation of ZAG with clinical parameters

Next, we investigated the association of plasma ZAG levels with various anthropometric and biochemical parameters by using partial correlations. Plasma ZAG correlated negatively with markers of adiposity (WHR, BMI, FAT% and FFA, all *P* < 0.01) and glucose metabolic parameters (FBG, 2 h–BG, HbA1c, FIns and HOMA-β, all *P* < 0.01; Table 2). It also closely correlated with insulin resistance indices (decreased HOMA-IR) and blood pressure (all *P* < 0.01; Table 2). In addition, Circulating ZAG levels correlated positively with age, HOMA-β and HDL-C (all *P* < 0.01; Table 2). Importantly, circulating ZAG also correlated positively with plasma ADI levels, an insulin sensitizer and cardio-protective adipokine (*P* < 0.01; Table 2). However, there was no significant correlation between ZAG levels and TC, LDL-C and 2 h–Ins.In multiple stepwise regression analysis, only BMI, TC, HOMA-IR and ADI were independent related factors with circulating ZAG levels (Table 2). The multiple regression equation was: Y_ZAG_ = 4.012–0.037 × BMI + 0.060 × TC -0.046 × HOMA-IR + 0.013 × ADI **(** *R* = 0.750, *R* ^*2*^ = 0.563).

**Table 2:** Linear regression analysis of variables associated with circulating ZAG levels in the study population

Variable	Simple		Multiple	
	*r*	*P*-value	β	*P*-value
Age (year)	0.229	<0.001		
BMI (kg/m^2^)	−0.565	<0.001	−0.037	<0.001
FAT (%)	−0.213	<0.001		
WHR	−0.221	<0.001		
SBP (mmHg)	−0.237	<0.001		
DBP (mmHg)	−0.094	0.039		
TC (mmol/L)	0.054	NS	0.060	<0.001
TG (mmol/L)	−0.215	<0.001		
HDL-C (mmol/L)	0.130	0.004		
LDL-C (mmol/L)	0.005	NS		
FFA (umol/L)	−0.137	0.002		
FBG (mmol/L)	−0.498	<0.001		
2 h–BG (mmol/L)	−0.475	<0.001		
FIns (mU/L)	−0.413	<0.001		
2 h–Ins (mU/L)	−0.047	NS		
HbA1c (%)	−0.574	<0.001		
HOMA-IR	−0.538	<0.001	−0.046	<0.001
HOMA-β	0.211	<0.001		
Adiponectin (μg/L)	0.610	<0.001	0.013	<0.001

In multiple linear regression analysis, values included for analysis were age, sex, BMI, WHR, BP, FBG, insulin, HOMA-IR, HOMA-β, FFA, total cholesterol, HDL-C, LDL-C, triglyceride and Adiponectin

### ZAG characteristics according to plasma ADI quartiles and MetS components

**Fig. 2:**
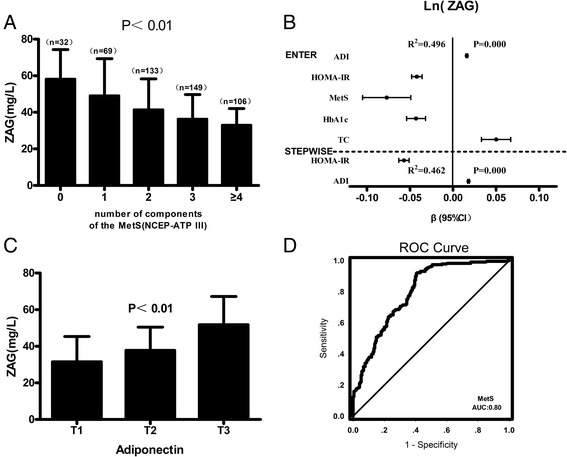
**a**: Circulating ZAG levels in relation to the number of MetS components; **b**: All factors and stepwise multiple regression analyses of the circulating ZAG and MetS in study population. The circles correspond to the regression coefficients (β) and the error bars indicate the 95% confidence interval of β. *R*_*2*_ = coefficient of determination; **c**: Least square means of circulating level of ZAG according to ADI tertiles in all the subjects; **d**: ROC curve analysis was performed for the prediction of MetS. Values are means ± SD

In order to understand the association between ZAG and ADI, the population was divided according to tertiles of ADI (T1: 0–26.52; T2, 26.53–31.75; T3, >31.75 μg/L). Age adjusted estimates of ZAG means showed significant association between levels of ZAG and ADI among all subgroups. Overall, subjects in the highest tertiles of ADI had the highest ZAG levels (Fig. 2c). To further explore the association of circulating ZAG levels with MetS, we stratified the mean levels of circulating ZAG by the number of components of the MetS. The result indicated that circulating ZAG reduced progressively with increasing number of the MetS components (*P* for trend <0.01) (Fig. 2a). Patientswith 0, 1, 2, 3or more component (s) of the MetS had reduced circulating ZAG levels of 58.3 ± 15.9; 49.2 ± 20.1; 41.5 ± 16.7; 36.4 ± 13.3; 33.1 ± 8.8 μg/L (mean ± SD), respectively (Fig. 2a).

### Binary logistic regression and ROC curves analysis

Binary logistic regression analysis showed that plasma ZAG concentrations were significantly associated with MetS even after controlling for anthropometric variables, lipidprofile and hormone levels (Table 3). When considering MetS patients and controls as a whole, regression analyses, including all-factor and stepwise models, showed that the main predictors of circulating ZAG levels were HOMA-IR, ADI, HbA1c, TC and MetS (Fig. 2b). Furthermore, when concentrations were analyzed both by a Row Mean Scores test and a Cochran-Armitage trend test, the reduced levels of circulating ZAG showed a significant linear trend and were independently associated with MetS (Table 4). To explore the predictive value of ZAG for MetS, we analyzed the receiver operating characteristic (ROC) curves of plasma ZAG. The results revealed that the best cutoff value for circulating ZAG to predict MetS was 45.2 mg/L (sensitivity 92%, specificity 59%, and AUC 0.80; Fig. 2d).

**Table 3:** Association of circulating ZAG with MetSin fully adjusted models

Model adjust	Mets		
	OR	95% CI	*P*-value
Age, SBP, DBP	0.953	0.939–0.968	<0.001
Age, SBP, DBP, WHR, BMI	0.970	0.953–0.987	0.001
Age, SBP, DBP, WHR, BMI, lipid profile	0.982	0.962–1.004	0.103

Results of multivariate logistic regression analysis are presented as the odds ratio (OR) of being in MetS status decrease in circulating. *BMI* body mass index, *WHR* waist-to-hip, *SBP* systolic blood pressure, *DBP* diastolic blood pressure; lipid profile, including total cholesterol, triglyceride, LDL- and HDL-cholesterol

**Table 4:** Row mean scores and Cochran–Armitage trend testof the impact of plasma ZAG level on MetS

	Mets	
	χ^2^	*P*-value
ROW Mean Scores Test	56.4118	<0.001
Cochran-Armitage Test	−32.5801	<0.001

The circulating ZAG levels of all subjects were cut-offand adjusted for age, sex, BMI, WHR, SBP, DBP, TC, TG,LDL-C and HDL-C

## Discussion

One study has revealed that MetS patients have higher serum ZAG levels than individuals without MetS and circulating levels of ZAG elevated progressively with an increasing number of MetS components [17]. However, as with most new discoveries, these findings need to be reproduced and confirmed. Therefore, we consider it important to examine a large population to determine whether circulating ZAG correlates with MetS, lipid profiles and ADI (another adipocyte-secreted hormone that has important associations with MetS). In the current study, we found that circulating ZAG levels were significantly lower in subjects with MetS than in those without MetS or in subjects with a higher number of MetS components such as IR and central obesity. We speculate that 1) the reduction in plasma ZAG concentration in MetS patients might be due to a defensive response, which may represent an ability to adapt to metabolic disturbance; 2) decreasing circulating ZAG might also suggest an increasing consumption of antagonizing metabolic stress, such as hyperglycemia and hyperlipidemia; 3) dysmetabolism might lead to the dysregulation of ZAG in biosynthesis and release. These factors need to be further clarified with in vivo and *vitro* studies. However, to our surprise, these results are not in agreement with previous findings in which circulating ZAG was found to be elevated in patients with MetS [17]. The differences in study design, including in patients election (e.g., aging, obesity or lean, glycaemia and lipidemia levels, sample size, agent treatment, other diseases, and complications) and experimental conditions likely contribute to the disparity. Furthermore, derangements in glucose homeostasis or analtered lipid profile may exert influence on the release of ZAG from body tissues. In the current study, MetS patients were newly diagnosed and were not treated with oral agents or special diets. These factors may in fact strengthen the analysis in our study. However, the reduced circulating ZAG levels in MetS subjects need to be further investigated.

The present study data also indicates that circulating ZAG correlates positively with age, HDL-C, and HOMA-β and negatively with parameters of adiposity (WHR, FAT% and BMI), IR (increased fasting insulin and HOMA-IR), dyslipidemia (increased TG and FFA), DBP, and SBP. Lower ZAG levels in MetS patients were associated with parameters of glucose metabolism (increasing FBG, 2 h–BG and HbA1c). In addition, it has been reported that ADI is a well-established marker for IR. Therefore, it is important to verify the association between ZAG and ADI. In the current study, we found that circulating ZAG levels correlated positively with plasma ADI levels in our study population, further confirming that ZAG is related to IR and may be a sensitizer. These results also suggest that circulating ZAG levels reflect the changes of MetS components and support the hypothesis that ZAG should be considered a marker of IR directly involved in the homeostasis of glucose and lipid metabolism.

To determine the predictive value of ZAG for MetS, we analyzed the ROC curves of circulating ZAG and MetS. The results showed that ZAG may be a good predictor for the diagnosis of MetS. The optimal cutoff value of ZAG for identifying individuals with MetS was also determined. Our study also has some limitations. First, a cross-sectional design limits any firm conclusion about the possible causative role of ZAG in MetS. Longitudinal intervention studies and future investigation are necessary. Secondly, our sample consisted entirely of Chinese people. Therefore, the extrapolation of these results to other ethnic groups should be undertaken with caution. Third, ZAG was not a prespecified end point of recruited MetS subjects in the study, and measurements of ZAG were made on stored samples, although the samples were relatively fresh. Furthermore, the current analyses are based on single measurements of circulating ZAG, which may not reflect ZAG levels over time. Serial changes in circulation need to be measured at different stages of MetS. Nevertheless, the strengths of this study include 1) newly diagnosed MetS subjects prevent pharmacotherapy and other confounding variables or complications; 2) the association between circulating ZAG and MetS is investigated; 3) the predictive value of circulating ZAG for MetS is evaluated; 4) relatively large sample size.

## Conclusion

In summary, our data showed that circulating ZAG levels are decreased in patients with MetS. Circulating ZAG decreased progressively with an increasing number of MetS components and it was associated with ADI levels, suggesting that ZAG is related to IR and MetS and may be an insulin sensitizer. Therefore, ZAG may be a useful marker for the prediction of MetS.

## Abbreviations

2 h–BG: 2-h blood glucose after glucose overload
2 h–Ins: 2-h plasma insulin after glucose overload
ADI: Adiponectin
BMI: Body mass index
Fat%: The percentage of fat in vivo
FBG: Fasting blood glucose
FFA: Free fatty acid
HDL-C: High-density lipoprotein cholesterol
HOMA-IR: Homeostasis model assessment of insulin resistance
HOMA-β: HOMA β cell insulin secretion index
ROC: Receiver operating characteristic
WHR: Waist hip ratio
